# Pentyl (*E*)-3-(3,4-dihy­droxy­phen­yl)acrylate

**DOI:** 10.1107/S1600536811040499

**Published:** 2011-10-08

**Authors:** Jun Wang, Shuangshuang Gu, Leixia Zhang, Fuan Wu, Xijie Guo

**Affiliations:** a School of Biological and Chemical Engineering, Jiangsu University of Science and Technology, Zhenjiang 212003, People’s Republic of China; b Sericultural Research Institute, Chinese Academy of Agricultural Sciences, Zhenjiang 212018, People’s Republic of China

## Abstract

In the mol­ecule of the title compound, C_14_H_18_O_4_, the C=C double bond is in an *E* configuration. The mol­ecule is almost planar (r.m.s. deviation of all non-H atoms = 0.04 Å). An intra­molecular O—H⋯O hydrogen bond occurs. In the crystal, inter­molecular O—H⋯O inter­actions link the mol­ecules into ribbons extending in [110].

## Related literature

For general background to the biological activity of caffeic acid and its esters, see: Uwai *et al.* (2008 ▶); Buzzi *et al.* (2009 ▶); For the preparation, see: Xia *et al.* (2006 ▶); Son *et al.* (2011 ▶). For bond-length data, see: Allen *et al.* (1987 ▶).
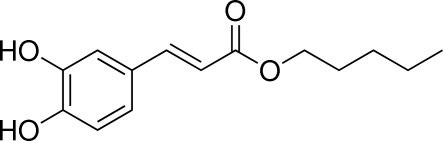


## Experimental

### 

#### Crystal data

C_14_H_18_O_4_
*M* *_r_* = 250.28
Triclinic, 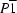

*a* = 5.3070 (11) Å
*b* = 10.567 (2) Å
*c* = 11.816 (2) Å
α = 90.96 (3)°
β = 91.84 (3)°
γ = 98.60 (3)°
*V* = 654.7 (2) Å^3^
*Z* = 2
Mo *K*α radiation
μ = 0.09 mm^−1^
*T* = 293 K
0.30 × 0.20 × 0.10 mm

#### Data collection

Enraf–Nonius CAD-4 diffractometer
Absorption correction: ψ scan (North *et al.*, 1968 ▶) *T*_min_ = 0.973, *T*_max_ = 0.991
2703 measured reflections
2419 independent reflections
1627 reflections with *I* > 2σ(*I*)
*R*_int_ = 0.023
3 standard reflections every 200 reflections  intensity decay: 1%

#### Refinement

*R*[*F* ^2^ > 2σ(*F* ^2^)] = 0.057
*wR*(*F* ^2^) = 0.169
*S* = 1.00
2419 reflections
163 parameters
H-atom parameters constrained
Δρ_max_ = 0.17 e Å^−3^
Δρ_min_ = −0.19 e Å^−3^

### 

Data collection: *CAD-4 EXPRESS* (Enraf–Nonius, 1994 ▶); cell refinement: *CAD-4 EXPRESS*; data reduction: *XCAD4* (Harms & Wocadlo, 1995 ▶); program(s) used to solve structure: *SHELXS97* (Sheldrick, 2008 ▶); program(s) used to refine structure: *SHELXL97* (Sheldrick, 2008 ▶); molecular graphics: *SHELXTL* (Sheldrick, 2008 ▶); software used to prepare material for publication: *PLATON* (Spek, 2009 ▶).

## Supplementary Material

Crystal structure: contains datablock(s) I, global. DOI: 10.1107/S1600536811040499/gw2109sup1.cif

Structure factors: contains datablock(s) I. DOI: 10.1107/S1600536811040499/gw2109Isup2.hkl

Supplementary material file. DOI: 10.1107/S1600536811040499/gw2109Isup3.cml

Additional supplementary materials:  crystallographic information; 3D view; checkCIF report

## Figures and Tables

**Table 1:** Hydrogen-bond geometry (Å, °)

*D*—H⋯*A*	*D*—H	H⋯*A*	*D*⋯*A*	*D*—H⋯*A*
O1—H1*B*⋯O2	0.82	2.30	2.738 (2)	114
O1—H1*B*⋯O2^i^	0.82	2.15	2.840 (2)	142
O2—H2*A*⋯O3^ii^	0.82	1.98	2.800 (2)	173
C5—H5*A*⋯O3^ii^	0.93	2.52	3.230 (3)	133

Symmetry codes: (i) 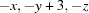; (ii) 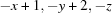.

**Fig. 1.**
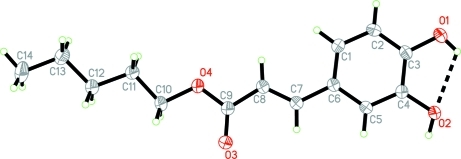
The molecular structure of the title molecule, with the atom numbering scheme. Displacement ellipsoids are drawn at 30% probability levels.

**Fig. 2.**
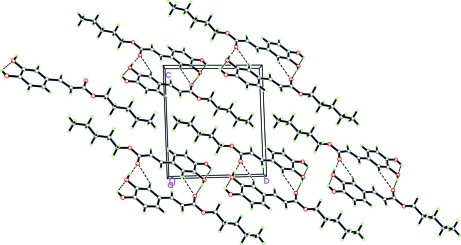
A partial packing diagram. Hydrogen bonds are shown as dashed lines.

## supplementary crystallographic information

### Comment

Caffeic acid and its esters have been a research hot spot for a long time. These compounds are known to show a variety of biological effects such as anti-tumor, anti-oxidant, and anti-inflammatory activities (Uwai *et al.*, 2008; Buzzi *et al.*, 2009). As a part of our studies into the synthesis of caffeic acid derivatives, the title compound (1) pentyl (*E*)-3-(3,4-dihydroxyphenyl)acrylate was synthesized (Xia *et al.* (2006); Son *et al.* (2011)). We report herein the crystal structure of the title compound.

The molecule of (I) has an *E* configuration (Fig. 1); All non-H atoms of (I) are almost coplanar, with a root mean square deviating from the least-squares plane of 0.04 A°. The bond lengths (Allen *et al.*, 1987) and angles are within normal ranges.

In the crystal structure, hydroxy groups contribute to intermolecular O—H···O interactions (Table 1) link the molecules into ribbons extended in the [110] direction (Fig. 2), in which they may be effective in the stabilization of thestructure. On the other hand, the intramolecular O—H···O H-bond also contribute to the stability of the molecular configuration (Fig. 1 and Table 1).

### Experimental

Esterification of caffeic acid with amyl alcohol was performed in a column (inner diameter= 15 mm, length = 200 mm). Caffeic acid (8.95 g, 0.05 mol) was dissolved in amyl alcohol (100 ml). The mixture was stirred at 80°C for 60 minutes and fed from the top of the reactor with syringe pumps. The feed rate of the mixture was fixed at 10.0 ml/h. Cation exchange resin CD-552 particles(5 g) and molecular sieve(5 g) were packed into the middle of the reactor and glass beads of 2 mm in diameter were loaded into the rest of the column. The reaction temperature continued at 90°C for 20 h. The mixture was evaporated to dryness and followed by the addition of ethanol and extracted with chloroform three times. The chloroform extract was dried over evaporated to give a solid residue, and dissolved in ethanol/petroleum ether (1:1) to crystal. The solution was filtered and concentrated to yield a brown crystalline product (5.3 g, 59.2%). Recrystallization from ethanol gave colourless crystal.

### Refinement

The H atoms were placed in calculated positions (O—H = 0.82 A ° and C—H = 0.93–0.97 A °) and constrained to ride on their parent atoms, with *U*_iso_(H) = 1.2 or 1.5Ueq(O,*C*).

### Crystal data

**Table:**

C_14_H_18_O_4_	*Z* = 2
*M**_r_* = 250.28	*F*(000) = 268
Triclinic, *P*1	*D*_x_ = 1.270 Mg m^−^^3^
Hall symbol: -P 1	Mo *K*α radiation, λ = 0.71073 Å
*a* = 5.3070 (11) Å	Cell parameters from 25 reflections
*b* = 10.567 (2) Å	θ = 9–13°
*c* = 11.816 (2) Å	µ = 0.09 mm^−^^1^
α = 90.96 (3)°	*T* = 293 K
β = 91.84 (3)°	Block, colourless
γ = 98.60 (3)°	0.30 × 0.20 × 0.10 mm
*V* = 654.7 (2) Å^3^	

### Data collection

**Table:**

Enraf–Nonius CAD-4 diffractometer	1627 reflections with *I* > 2σ(*I*)
Radiation source: fine-focus sealed tube	*R*_int_ = 0.023
graphite	θ_max_ = 25.4°, θ_min_ = 1.7°
ω/2θ scans	*h* = 0→6
Absorption correction: ψ scan (North *et al.*, 1968)	*k* = −12→12
*T*_min_ = 0.973, *T*_max_ = 0.991	*l* = −14→14
2703 measured reflections	3 standard reflections every 200 reflections
2419 independent reflections	intensity decay: 1%

### Refinement

**Table:**

Refinement on *F*^2^	Primary atom site location: structure-invariant direct methods
Least-squares matrix: full	Secondary atom site location: difference Fourier map
*R*[*F*^2^ > 2σ(*F*^2^)] = 0.057	Hydrogen site location: inferred from neighbouring sites
*wR*(*F*^2^) = 0.169	H-atom parameters constrained
*S* = 1.00	*w* = 1/[σ^2^(*F*_o_^2^) + (0.1*P*)^2^ + 0.040*P*] where *P* = (*F*_o_^2^ + 2*F*_c_^2^)/3
2419 reflections	(Δ/σ)_max_ < 0.001
163 parameters	Δρ_max_ = 0.17 e Å^−^^3^
0 restraints	Δρ_min_ = −0.19 e Å^−^^3^

### Special details

**Table:**

Geometry. All e.s.d.'s (except the e.s.d. in the dihedral angle between two l.s. planes) are estimated using the full covariance matrix. The cell e.s.d.'s are taken into account individually in the estimation of e.s.d.'s in distances, angles and torsion angles; correlations between e.s.d.'s in cell parameters are only used when they are defined by crystal symmetry. An approximate (isotropic) treatment of cell e.s.d.'s is used for estimating e.s.d.'s involving l.s. planes.
Refinement. Refinement of *F*^2^ against ALL reflections. The weighted *R*-factor *wR* and goodness of fit *S* are based on *F*^2^, conventional *R*-factors *R* are based on *F*, with *F* set to zero for negative *F*^2^. The threshold expression of *F*^2^ > σ(*F*^2^) is used only for calculating *R*-factors(gt) *etc*. and is not relevant to the choice of reflections for refinement. *R*-factors based on *F*^2^ are statistically about twice as large as those based on *F*, and *R*- factors based on ALL data will be even larger.

### Fractional atomic coordinates and isotropic or equivalent isotropic displacement parameters (Å^2^)

**Table:**

	*x*	*y*	*z*	*U*_iso_*/*U*_eq_	
O1	−0.2441 (3)	1.39149 (14)	0.11914 (14)	0.0617 (5)	
H1B	−0.1743	1.4410	0.0731	0.093*	
C1	−0.1068 (4)	1.08131 (19)	0.20524 (17)	0.0469 (5)	
H1A	−0.1712	1.0179	0.2547	0.056*	
O2	0.1576 (3)	1.36636 (13)	−0.01491 (12)	0.0535 (4)	
H2A	0.2656	1.3441	−0.0559	0.080*	
C2	−0.2175 (4)	1.1903 (2)	0.19717 (18)	0.0506 (6)	
H2B	−0.3549	1.2002	0.2416	0.061*	
C3	−0.1266 (4)	1.28540 (18)	0.12364 (17)	0.0435 (5)	
O3	0.4807 (3)	0.73067 (15)	0.14536 (14)	0.0699 (6)	
O4	0.2176 (3)	0.65259 (13)	0.27822 (13)	0.0578 (5)	
C4	0.0776 (4)	1.26997 (18)	0.05777 (16)	0.0408 (5)	
C5	0.1902 (4)	1.16118 (18)	0.06704 (16)	0.0408 (5)	
H5A	0.3293	1.1521	0.0233	0.049*	
C6	0.0999 (4)	1.06446 (18)	0.14062 (16)	0.0396 (5)	
C7	0.2247 (4)	0.95104 (18)	0.14637 (17)	0.0453 (5)	
H7A	0.3589	0.9487	0.0980	0.054*	
C8	0.1716 (4)	0.85117 (19)	0.21148 (17)	0.0493 (6)	
H8A	0.0415	0.8506	0.2624	0.059*	
C9	0.3082 (4)	0.74206 (19)	0.20677 (17)	0.0457 (5)	
C10	0.3335 (4)	0.53739 (19)	0.28150 (19)	0.0519 (6)	
H10A	0.5153	0.5582	0.2982	0.062*	
H10B	0.3079	0.4919	0.2091	0.062*	
C11	0.2072 (5)	0.4569 (2)	0.37279 (18)	0.0525 (6)	
H11A	0.0261	0.4364	0.3542	0.063*	
H11B	0.2270	0.5057	0.4437	0.063*	
C12	0.3173 (4)	0.3342 (2)	0.38791 (18)	0.0512 (6)	
H12A	0.2967	0.2858	0.3169	0.061*	
H12B	0.4987	0.3553	0.4055	0.061*	
C13	0.1962 (5)	0.2508 (2)	0.4799 (2)	0.0664 (7)	
H13A	0.0162	0.2262	0.4609	0.080*	
H13B	0.2109	0.2998	0.5506	0.080*	
C14	0.3177 (7)	0.1313 (3)	0.4962 (3)	0.0930 (10)	
H14A	0.2336	0.0813	0.5549	0.140*	
H14B	0.4949	0.1551	0.5172	0.140*	
H14C	0.3016	0.0818	0.4268	0.140*	

### Atomic displacement parameters (Å^2^)

**Table:**

	*U*^11^	*U*^22^	*U*^33^	*U*^12^	*U*^13^	*U*^23^
O1	0.0648 (10)	0.0488 (9)	0.0805 (11)	0.0307 (8)	0.0265 (8)	0.0173 (8)
C1	0.0505 (13)	0.0405 (11)	0.0512 (12)	0.0086 (9)	0.0125 (10)	0.0095 (9)
O2	0.0618 (10)	0.0405 (8)	0.0644 (9)	0.0217 (7)	0.0251 (8)	0.0162 (7)
C2	0.0468 (12)	0.0504 (13)	0.0587 (13)	0.0164 (10)	0.0180 (10)	0.0057 (10)
C3	0.0438 (12)	0.0371 (11)	0.0522 (12)	0.0139 (9)	0.0048 (9)	0.0010 (9)
O3	0.0806 (12)	0.0553 (10)	0.0841 (12)	0.0321 (8)	0.0460 (10)	0.0256 (8)
O4	0.0741 (11)	0.0403 (8)	0.0658 (10)	0.0227 (7)	0.0301 (8)	0.0172 (7)
C4	0.0456 (11)	0.0339 (10)	0.0439 (11)	0.0081 (9)	0.0059 (9)	0.0039 (8)
C5	0.0408 (11)	0.0383 (11)	0.0456 (11)	0.0116 (9)	0.0091 (9)	0.0025 (9)
C6	0.0427 (11)	0.0342 (10)	0.0430 (10)	0.0089 (8)	0.0040 (9)	0.0008 (8)
C7	0.0488 (12)	0.0400 (11)	0.0492 (11)	0.0114 (9)	0.0116 (10)	0.0039 (9)
C8	0.0573 (13)	0.0371 (11)	0.0569 (13)	0.0147 (10)	0.0189 (11)	0.0065 (10)
C9	0.0515 (13)	0.0392 (11)	0.0482 (11)	0.0099 (9)	0.0112 (10)	0.0048 (9)
C10	0.0640 (14)	0.0362 (11)	0.0601 (13)	0.0190 (10)	0.0157 (11)	0.0082 (10)
C11	0.0644 (15)	0.0430 (12)	0.0534 (12)	0.0151 (10)	0.0181 (11)	0.0072 (10)
C12	0.0580 (14)	0.0430 (12)	0.0555 (13)	0.0142 (10)	0.0090 (11)	0.0084 (10)
C13	0.0863 (19)	0.0551 (14)	0.0610 (14)	0.0161 (13)	0.0184 (13)	0.0158 (11)
C14	0.126 (3)	0.0666 (17)	0.095 (2)	0.0349 (17)	0.0191 (19)	0.0386 (15)

### Geometric parameters (Å, °)

**Table:**

O1—C3	1.363 (2)	C7—H7A	0.9300
O1—H1B	0.8200	C8—C9	1.452 (3)
C1—C2	1.372 (3)	C8—H8A	0.9300
C1—C6	1.388 (3)	C10—C11	1.498 (3)
C1—H1A	0.9300	C10—H10A	0.9700
O2—C4	1.371 (2)	C10—H10B	0.9700
O2—H2A	0.8200	C11—C12	1.512 (3)
C2—C3	1.382 (3)	C11—H11A	0.9700
C2—H2B	0.9300	C11—H11B	0.9700
C3—C4	1.382 (3)	C12—C13	1.509 (3)
O3—C9	1.205 (2)	C12—H12A	0.9700
O4—C9	1.324 (2)	C12—H12B	0.9700
O4—C10	1.444 (2)	C13—C14	1.513 (3)
C4—C5	1.377 (3)	C13—H13A	0.9700
C5—C6	1.393 (3)	C13—H13B	0.9700
C5—H5A	0.9300	C14—H14A	0.9600
C6—C7	1.455 (3)	C14—H14B	0.9600
C7—C8	1.318 (3)	C14—H14C	0.9600
			
C3—O1—H1B	109.5	O4—C10—C11	106.74 (16)
C2—C1—C6	120.89 (18)	O4—C10—H10A	110.4
C2—C1—H1A	119.6	C11—C10—H10A	110.4
C6—C1—H1A	119.6	O4—C10—H10B	110.4
C4—O2—H2A	109.5	C11—C10—H10B	110.4
C1—C2—C3	120.64 (18)	H10A—C10—H10B	108.6
C1—C2—H2B	119.7	C10—C11—C12	112.26 (17)
C3—C2—H2B	119.7	C10—C11—H11A	109.2
O1—C3—C2	118.08 (17)	C12—C11—H11A	109.2
O1—C3—C4	122.54 (18)	C10—C11—H11B	109.2
C2—C3—C4	119.38 (18)	C12—C11—H11B	109.2
C9—O4—C10	117.71 (15)	H11A—C11—H11B	107.9
O2—C4—C5	123.18 (17)	C13—C12—C11	113.87 (18)
O2—C4—C3	116.98 (17)	C13—C12—H12A	108.8
C5—C4—C3	119.85 (18)	C11—C12—H12A	108.8
C4—C5—C6	121.30 (17)	C13—C12—H12B	108.8
C4—C5—H5A	119.4	C11—C12—H12B	108.8
C6—C5—H5A	119.4	H12A—C12—H12B	107.7
C1—C6—C5	117.94 (18)	C12—C13—C14	112.6 (2)
C1—C6—C7	123.06 (18)	C12—C13—H13A	109.1
C5—C6—C7	119.01 (17)	C14—C13—H13A	109.1
C8—C7—C6	128.16 (19)	C12—C13—H13B	109.1
C8—C7—H7A	115.9	C14—C13—H13B	109.1
C6—C7—H7A	115.9	H13A—C13—H13B	107.8
C7—C8—C9	122.58 (19)	C13—C14—H14A	109.5
C7—C8—H8A	118.7	C13—C14—H14B	109.5
C9—C8—H8A	118.7	H14A—C14—H14B	109.5
O3—C9—O4	122.77 (19)	C13—C14—H14C	109.5
O3—C9—C8	125.58 (19)	H14A—C14—H14C	109.5
O4—C9—C8	111.64 (17)	H14B—C14—H14C	109.5
			
C6—C1—C2—C3	0.5 (3)	C4—C5—C6—C7	179.50 (18)
C1—C2—C3—O1	179.97 (19)	C1—C6—C7—C8	−1.5 (4)
C1—C2—C3—C4	0.1 (3)	C5—C6—C7—C8	178.6 (2)
O1—C3—C4—O2	−0.8 (3)	C6—C7—C8—C9	178.43 (19)
C2—C3—C4—O2	179.06 (18)	C10—O4—C9—O3	0.1 (3)
O1—C3—C4—C5	179.26 (18)	C10—O4—C9—C8	179.17 (18)
C2—C3—C4—C5	−0.8 (3)	C7—C8—C9—O3	0.3 (4)
O2—C4—C5—C6	−178.85 (17)	C7—C8—C9—O4	−178.7 (2)
C3—C4—C5—C6	1.0 (3)	C9—O4—C10—C11	176.82 (18)
C2—C1—C6—C5	−0.4 (3)	O4—C10—C11—C12	−178.17 (18)
C2—C1—C6—C7	179.72 (19)	C10—C11—C12—C13	179.56 (19)
C4—C5—C6—C1	−0.4 (3)	C11—C12—C13—C14	−177.7 (2)

### Hydrogen-bond geometry (Å, °)

**Table:**

*D*—H···*A*	*D*—H	H···*A*	*D*···*A*	*D*—H···*A*
O1—H1B···O2	0.82	2.30	2.738 (2)	114
O1—H1B···O2^i^	0.82	2.15	2.840 (2)	142
O2—H2A···O3^ii^	0.82	1.98	2.800 (2)	173
C5—H5A···O3^ii^	0.93	2.52	3.230 (3)	133

Symmetry codes:  (i) −*x*, −*y*+3, −*z*; (ii) −*x*+1, −*y*+2, −*z*.
